# Conservation and Convergence of Colour Genetics: *MC1R* Mutations in *brown* Cavefish

**DOI:** 10.1371/journal.pgen.1000388

**Published:** 2009-02-20

**Authors:** Nicholas I. Mundy

**Affiliations:** Department of Zoology, University of Cambridge, Cambridge, United Kingdom; Stanford University School of Medicine, United States of America

One of the most striking observations in nature is when similar phenotypes appear independently, such as wings in birds and bats, or melanism in moths and mice. These examples of so-called convergent evolution naturally lead us to ponder the question of genetic repeatability, i.e., the extent to which similar phenotypes that evolved in parallel share the same genetic mechanisms. Cave-dwelling organisms provide an attractive system for studying genetic repeatability, since populations in geographically isolated caves often undergo striking convergent evolution in response to the drastically altered environment, with reduced pigmentation and vision being particularly common phenotypes. In a paper recently published in *PLoS Genetics* [1], Gross et al. find that different mutations at the same locus, *MC1R* (*Melanocortin-1 receptor*), underlie the parallel evolution of reduction of pigmentation in a teleost fish, the Mexican cave tetra *Astyanax mexicanus*. The *MC1R* has been widely implicated in the evolution of colouration in birds and mammals, and the current results add to a growing body of literature showing that genetic repeatability in evolution is surprisingly common, although by no means pervasive (e.g., [2]). A role for *MC1R* in teleost pigmentation is also interesting in the light of differences in pigmentary biology between homeothermic amniotes (mammals and birds) and other vertebrates.

The authors studied the *brown* mutation, a recessive mutation in which *A. mexicanus* have paler skin and eyes than fish from surface-dwelling populations, and found that both the number of melanin-producing pigment cells (melanophores) and their melanin content were decreased in the dorsal skin. Complementation tests had previously shown that the *brown* mutation was probably at the same locus in several isolated caves in Northeastern Mexico, including Pachón, Yerbaniz/Japonés, Curva, and Piedras. Quantitative trait locus (QTL) mapping on an F_2_ derived from a surface× Pachón cave *brown* cross identified a single peak in logarithm of the odds (LOD) score in the genome that contained the *MC1R* locus based on comparative mapping to zebrafish. The authors found that *brown* cavefish from Pachón carried an early frameshift in *MC1R* (Δ24,25), whereas the *brown* mutation in the Yerbaniz/Japonés population carried a missense alteration, R164C—remarkably, the identical mutation at the homologous MC1R residue in humans causes a loss-of-function mutation that gives rise to red hair and fair skin [3]. In contrast, there were no amino-acid–changing mutations in several other populations, including Curva and Piedras, suggesting that *cis*-regulatory mutations in *MC1R* may be involved in these cases. Confirmation that these *MC1R* mutations have functional consequences came from experiments exploiting gene knockdown technology in zebrafish. Consistent with a hypothesis of reduced or absent MC1R function in the two coding mutations, zebrafish treated with a *MC1R* morpholino had reduced pigmentation that could be rescued by wild-type *Astyanax MC1R*, but not by the Δ24,25 or R164C variants.

These results are interesting because, up until now, the sole function of *MC1R* in fish and other poikilotherms was considered to be short term physiological colour change to match the environment, as in frogs and chameleons [4]. MC1R is a seven-transmembrane G-protein–coupled receptor expressed by melanophores that, when activated by the hormone MSH (melanocyte-stimulating hormone), causes intracellular dispersion of membrane-bound pigment granules (melanosomes) within the melanophore leading to darker colouration. This process is reversed in response to a second hormone, MCH (melanin-concentrating hormone) [4]. The results from *A. mexicanus* show that *MC1R* can also function earlier in the pigmentation pathway in teleosts to affect both melanophore number and the amount of melanin in each melanophore. It will be interesting to investigate these novel functions in more detail, including how they relate to other genes involved in pigment cell development in fish [5].

The comparison with MC1R function in mammals and birds is instructive. In these lineages, pigment cells slowly transfer melanin granules to adjacent keratinocytes using a different set of biochemical and cell biologic pathways, and these cells are termed melanocytes to reflect this difference. Consequently, mammals and birds are unable to change their colour rapidly. Instead, a major function of MC1R in mammals and birds is to act as a switch between synthesis of dark eumelanin and phaeomelanin, a pale or reddish melanin that is apparently absent from teleosts. Thus, evolution of dark/pale colouration has involved *MC1R* mutations repeatedly not only in birds and mammals [6],[7] (and probably also reptiles [8]), but now also in fish [1], in spite of many differences in mechanistic detail. An interesting difference, however, is that the MC1R variants in cavefish affect eye colour as well as body colour, whereas MC1R effects on eye colour have never been described in birds and mammals. More broadly, although colouration in fish (in contrast to mammals) is determined to a great extent by migration, proliferation, and cell–cell interactions among melanophores and other pigment cells [9], the present study adds to other reports showing a surprising amount of conservation in genes underlying melanin-based colouration among fish and mammals [10],[11].

What do the results say about the mechanisms of evolution in cave organisms? The evolutionary forces acting on loss-of-function phenotypes such as *brown* are hotly debated. Potential explanations for the rise in frequency of such phenotypes fall chiefly into three categories: (i) purely neutral, i.e., arising from genetic drift and/or inbreeding; (ii) direct selection for the loss-of-function phenotype, e.g., because of energetic constraints; and (iii) indirect selection on the loss-of-function phenotype arising as a correlated response to selection on a second trait controlled either by pleiotropic action of the same locus or by a closely linked locus. Assessing the relative importance of these mechanisms for *brown* is made complex but more intriguing by the presence of a second and more dramatic colour phenotype—albinism—which the same lab has shown to be caused by independent mutations in the *OCA2* locus [12]. Albinism masks (i.e., is epistatic to) the expression of *brown*, and wild populations contain differing proportions of albino and *brown* individuals. The Yerbaniz/Japonés population is fixed for albinism, so it could be that the *brown* mutation it contains (the R164C variant) has never been expressed, in which case neutral causes would be most likely. In contrast, the Pachón population has a mixture of both phenotypes. The most one can speculate here is that because *MC1R* and *OCA2* are unlinked, it would be surprising if the third mechanism was acting on both loci. From this perspective, it would be interesting to perform population genetic studies in this system to determine the relative importance of genetic drift and selection in loss of colouration and investigate the relative ages of the mutations at the different loci.
